# Characterization of a Novel *GATA4* Missense Variant p.Gly303Trp in a Family with Septal Heart Defects and Pulmonary Stenosis

**DOI:** 10.3390/ijms26104931

**Published:** 2025-05-21

**Authors:** Marco Fabiani, Costanza Zangheri, Antonella Cima, Francesca Monaco, Chiara Ali’, Maria Antonietta Barone, Antonella Viola, Alvaro Mesoraca, Katia Margiotti, Claudio Giorlandino

**Affiliations:** 1 Human Genetics Lab, Altamedica Main Centre, Viale Liegi 45, 00198 Rome, Italy; marco.fabiani@artemisia.it (M.F.); costanza.zangheri@artemisia.it (C.Z.); antonella.cima@artemisia.it (A.C.); francesca.monaco@artemisia.it (F.M.); chiaa.ali98@gmail.com (C.A.); mariantoniettabarone@libero.it (M.A.B.); antonellaviola@libero.it (A.V.); alvaro.mesoraca@artemisia.it (A.M.); claudio.giorlandino@artemisia.it (C.G.); 2 Department of Prenatal Diagnosis, Altamedica, Fetal-Maternal Medical Centre, Viale Liegi 45, 00198 Rome, Italy

**Keywords:** congenital heart disease ventricular septal defect, atrial septal defects, pulmonary stenosis, *GATA4*, whole exome sequencing

## Abstract

Congenital heart disease (CHD) represents a prevalent group of structural cardiac anomalies often associated with alterations in key transcription factors including *NKX2-5*, *TBX5*, and, particularly, *GATA4*. *GATA4* is a zinc finger transcription factor essential for regulating genes involved in cardiogenesis. Here, we report the identification of a novel heterozygous missense variant in *GATA4* (NM_002052.5:c.907G>T, p.Gly303Trp) in a family with a history of CHD. The proband, exhibiting ventricular septal defect (VSD) and pulmonary stenosis, was referred for genetic evaluation after recurrent spontaneous abortions occurred in their partner. In addition, the mother of the proband has a history of atrial septal defect (ASD) with pulmonary stenosis, which suggests a familial inheritance pattern.

## 1. Introduction

Congenital heart disease (CHD) comprises a group of structural abnormalities resulting from defective cardiac and major vessel development during embryogenesis. As the most common type of birth defect worldwide, CHD affects approximately one-third of all major congenital anomalies. Its global prevalence varies by region, ranging from 6.9 per 1000 live births in Europe to 9.3 per 1000 in Asia [1,2]. Among CHD subtypes, ventricular septal defect (VSD), atrial septal defect (ASD), patent ductus arteriosus (PDA), and pulmonary stenosis (PS) account for over 60% of cases [1]. These defects often involve abnormal communication between cardiac chambers, leading to hemodynamic shunting between the systemic and pulmonary circuits. Despite their high prevalence, the etiology of CHD remains poorly understood. Both intrinsic (genetic) and extrinsic (environmental) risk factors, such as maternal diabetes, maternal smoking, and exposure to teratogens, have been implicated in CHD [3], and the intricate processes of cardiac development further complicate the understanding of their pathogenesis.

In recent decades, advances in developmental biology and genomics have improved our understanding of the genetic underpinnings of CHD [4]. Transcription factors (TFs) play a pivotal role in the regulation of cardiac morphogenesis, with key factors such as *NKX2.5*, *TBX5*, *GATA4*, *BMP4*, and *HAND1* frequently implicated in CHD [5,6,7,8]. In addition to the well-established transcription factors, *STAT4* has been identified as a factor that can potentially influence gene expression in cardiac cells, particularly through inflammatory responses [9]. Among these, *GATA4* has been extensively studied, with over 100 known pathogenic or likely pathogenic variants associated with CHD.

*GATA4* encodes a zinc finger transcription factor located on chromosome 8p23.1-p22 and is essential for early cardiac and endodermal development. The human *GATA4* gene spans approximately 50 kilobases and comprises six exons. Pathogenic variants in *GATA4* have been primarily associated with septal defects, particularly ASD and VSD, but also with other cardiac malformations, underscoring its central role in cardiac development. Structurally, *GATA4* contains two highly conserved zinc finger domains (N-terminal ZNI and C-terminal ZNII), critical for DNA binding, protein–protein interactions, and nuclear localization. Variants in the C-terminal zinc finger, which also overlaps with the nuclear localization signal (NLS), can disrupt transcriptional regulation of target genes and lead to congenital anomalies. In this study, we report a novel heterozygous missense variant in *GATA4*, NM_002052.5:c.907G>T (p.Gly303Trp), identified in a family presenting with a spectrum of septal heart defects and pulmonary stenosis. The proband exhibits a history of perimembranous VSD and residual shunting, while his mother has ASD and required surgical correction for pulmonary stenosis. This report contributes to the growing body of evidence linking *GATA4* variants to familial CHD and emphasizes the relevance of integrating clinical and genetic data in the diagnostic process.

## 2. Results

**Figure 1:**
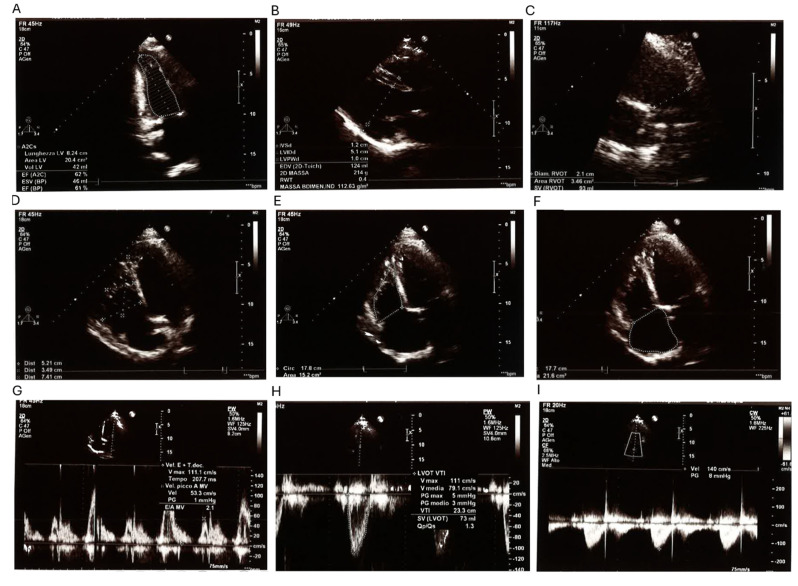
Comprehensive transthoracic echocardiographic assessment in a patient with a history of perimembranous ventricular septal defect (VSD) repair, patent foramen ovale (PFO) closure, and pulmonary artery plasty. (**A**) Apical two-chamber view showing a left ventricular (LV) end-diastolic volume of 42 mL, end-systolic volume of 46 mL, and preserved systolic function with ejection fraction (EF) of 62%. (**B**) Parasternal long axis view measuring interventricular septum (IVSd: 1.2 cm), LV internal diameter (LVIDd: 5.1 cm), posterior wall (LVPWd: 1.0 cm), and LV mass (214 g; indexed LV mass: 112.63 g/m^2^). (**C**) Right ventricular outflow tract (RVOT) view, with diameter of 2.1 cm, cross-sectional area of 3.46 cm^2^, and stroke volume of 93 mL. (**D**) Apical view of right chambers showing mild right atrial dilation (TD area: 30 cm^2^). (**E**) Four-chamber view demonstrating mild left atrial enlargement (area: 15.2 cm^2^) and eccentric tricuspid regurgitation directed toward the atrial septum. (**F**) Left atrial volume estimation (21.6 cm^3^). (**G**) Pulsed-wave Doppler of mitral inflow showing an E/A ratio of 2.1, suggestive of normal LV filling pressures. (**H**) LVOT Doppler showing VTI of 23.3 cm, stroke volume of 73 mL, and Qp/Qs ratio of 1.3, indicating a small residual left-to-right shunt. (**I**) Continuous-wave Doppler showing a pulmonary peak velocity of 140 cm/s and a pressure gradient of 8 mmHg, consistent with mild residual pulmonary valve insufficiency. Overall, systolic function is preserved, and there are no signs of pericardial effusion. Right-sided sections are mildly dilated with preserved contractility (TAPSE 25 mm, FAC 47%). The inferior vena cava is not dilated and shows normal collapsibility.

The proband is a 38-year-old male referred for genetic consultation due to a history of multiple first-trimester miscarriages in his partner. His medical history is significant for a surgically corrected perimembranous ventricular septal defect (VSD), which was repaired at 16 months of age. Despite early surgical intervention, a small residual shunt in the anterior portion of the membranous septum persists, as confirmed by current echocardiographic studies. A comprehensive transthoracic echocardiogram (Figure 1) showed a left ventricular end-diastolic volume (LV EDV) of 119 mL and an end-systolic volume (LV ESV) of 42 mL, placing the left ventricle at the upper limit of normal dimensions. Septal and parietal wall thicknesses were within reference values, and global systolic function was preserved (ejection fraction: 63%). Additional findings included mild left atrial enlargement and mild mitral valve regurgitation. The right heart chambers appeared mildly dilated but showed preserved contractility (TAPSE: 25 mm; FAC: 47%). Pulmonary artery systolic pressure (PAPs) was estimated at 39 mmHg, within the normal range. The inferior vena cava (IVC) was not dilated and exhibited normal collapsibility. No pericardial effusion was observed.

Electrocardiogram (Supplementary Figure S1) confirmed sinus rhythm at 57 bpm, with normal atrioventricular and intraventricular conduction. The electrical axis was normal, and there were no signs of repolarization abnormalities, ischemia, or ventricular hypertrophy. Overall, the proband presented a well-compensated hemodynamic profile, without signs of cardiac decompensation at the time of evaluation.

The proband’s 63-year-old mother also presented a history of congenital heart disease. At age 8, she underwent surgical closure of an ostium secundum atrial septal defect (ASD II) and pulmonary valvuloplasty (PVP) due to severe pulmonary stenosis (PS). At age 59, she was hospitalized for atrial fibrillation (AF); due to recurrent episodes, she recently underwent electroporation ablation. The familial occurrence of these cardiac anomalies, combined with the reproductive history of the proband, suggested a possible hereditary predisposition to congenital heart defects, prompting a comprehensive genetic evaluation. Initial genetic testing included standard cytogenetic karyotyping and chromosomal microarray analysis (aCGH), both of which returned negative results for pathogenic chromosomal structural rearrangements or copy number variations. Given the clinical suspicion of a monogenic etiology, whole exome sequencing (WES) was performed on the proband. WES analysis identified a novel heterozygous missense variant in the *GATA4* gene (NM_002052.5:c.907G>T; p.Gly303Trp). This variant leads to the substitution of glycine with tryptophan at position 303, a residue located within the nuclear localization signal (NLS) domain and highly conserved across vertebrate species. The G303W variant was subsequently confirmed by Sanger sequencing in the proband and in other available family members. Segregation analysis revealed that the same variant was present in his affected mother but absent in his asymptomatic sister, further supporting its association with disease. Additionally, the variant was not detected in his wife, who served as a negative control for variant inheritance in offspring-related analyses.

**Figure 2:**
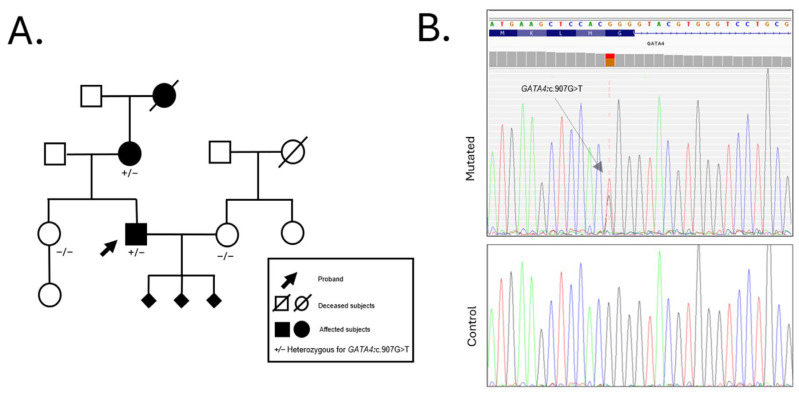
Familial segregation and molecular characterization of the GATA4 c.907G>T variant. (**A**) Pedigree of the family showing autosomal dominant inheritance. Genotypes for the GATA4 c.907G>T variant are shown under each tested individual: heterozygous carriers (+/−) are present among affected and unaffected subjects, while homozygous wild-type (−/−) individuals are unaffected. (**B**) Electropherogram showing Sanger sequencing validation of the GATA4 c.907G>T (p.Gly303Trp) variant. The upper panel represents the proband’s heterozygous mutation (arrow), while the lower panel shows a control subject with a wild-type sequence at the same position.

The family history is notable for a paternal grandfather who died of a myocardial infarction at the age of 80. Unfortunately, no genetic material was available from this individual, and thus, segregation of the variant could not be assessed in the paternal lineage. No other family members are reported to be clinically affected at the time of evaluation (Figure 2).

The NM_002052.5:c.907G>T variant has not been previously reported in the literature and is absent from major population databases, including gnomAD, ExAC, and 1000 Genomes. According to the American College of Medical Genetics and Genomics (ACMG) guidelines, this variant is currently classified as a Variant of Uncertain Significance (VUS), meeting the criteria PM2_Supporting (absence in control populations), PP3 (deleterious computational prediction), and PP2 (missense variant in a gene with low benign variation and known disease mechanism involving missense changes).

**Figure 3:**
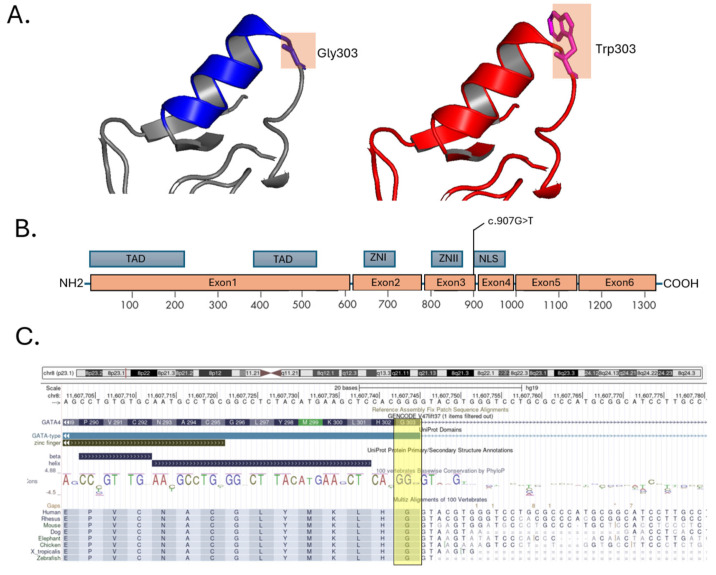
Structural and genomic characterization of the GATA4 c.907G>T (p.Gly303Trp) variant. (**A**) In silico protein modeling of the GATA4 zinc finger domain highlights the structural impact of the Gly303Trp substitution. The wild-type structure (left) shows Glycine at position 303, while the mutated structure (right) illustrates the steric hindrance of tryptophan residue, potentially affecting the spatial conformation and DNA-binding capacity. (**B**) Schematic representation of the GATA4 gene and protein domains. The c.907G>T variant is located within exon 4, corresponding to the nuclear localization signal (NLS) domain. The positions of the transcriptional activation domain (TAD), the two zinc finger domains (ZN I and ZN II), and the NLS are indicated. (**C**) UCSC Genome Browser Snapshot showing the position of the GATA4 variant (highlighted in yellow) in a highly conserved region across multiple vertebrate species. The alignment demonstrates evolutionary conservation of the mutated residue, supporting its functional relevance.

Multiple in silico prediction tools provided consistent evidence supporting a damaging effect of the *GATA4* p.Gly303Trp variant. The Combined Annotation Dependent Depletion (CADD) score was 31, placing this variant in the top 0.1% of potentially deleterious substitutions in the genome. The REVEL score was 0.869 (range 0–1), and PolyPhen-2 classified the variant as “probably damaging” with a score of 0.998. The PaPI (Pathogenicity Prediction for missense mutations) score was 1.0, further supporting a likely pathogenic role. Splicing impact analysis (dbscSNV) yielded an ADA score of 0.989 and a Random Forest score of 0.844, although the variant is located away from canonical splice sites. Importantly, the AlphaMissense deep learning model, which integrates structural and evolutionary context, classified the variant as “deleterious (strong)” with a score of 0.999. Cross-species alignment confirmed that Gly303 is strictly conserved in *GATA4* orthologs from mammals to lower vertebrates, reinforcing its functional relevance. *GATA4* encodes a zinc finger transcription factor with two zinc-binding motifs. The Gly303 residue lies adjacent to conserved cysteine residues critical for zinc coordination within the C-terminal zinc finger domain and is part of the basic region constituting the NLS (Figure 3). Its strategic position suggests that the substitution could interfere with nuclear localization, DNA binding, or protein–protein interactions required for transcriptional activity.

## 3. Discussion

The heart is the first organ to develop during vertebrate embryogenesis, with cardiac progenitor cells beginning to differentiate as early as day 15 of gestation. These cells organize into the cardiac crescent, a precursor structure essential for proper heart formation. This process is tightly regulated by multiple signaling pathways and transcriptional regulators, including NKX2.5 and GATA4, which are activated by endoderm-derived signals such as Cerberus, Bone Morphogenetic Proteins (BMPs), and Fibroblast Growth Factor 8 (FGF-8) [10,11]. Disruption of these molecular pathways can lead to congenital heart disease (CHD), one of the most common developmental disorders worldwide.

In this study, we report the identification of a novel heterozygous missense variant in *GATA4* (NM_002052.5:c.907G>T; p.Gly303Trp) in a family affected by septal heart defects and pulmonary stenosis. The proband, who carries this variant, presents with a surgically corrected perimembranous ventricular septal defect (VSD) and a residual interventricular shunt. His mother, who also carries the same variant, was surgically treated for an ostium secundum atrial septal defect (ASD II) and severe pulmonary stenosis. These findings support the hypothesis of a familial monogenic form of CHD involving gene *GATA4*. All GATA proteins share a highly conserved structural organization, which includes two zinc finger domains: the N-terminal zinc finger (ZN I) and the C-terminal zinc finger (ZN II). The C-terminal domain is essential for DNA binding and protein–protein interactions and overlaps with the nuclear localization signal (NLS) [12,13]. These domains are responsible for binding DNA and interacting with other transcription factors. It is involved in early heart tube formation, looping, chamber septation, and valve morphogenesis [4,14]. Pathogenic variants in *GATA4* are known to cause a wide spectrum of CHDs, with septal defects such as ASD and VSD being the most frequently reported phenotypes GATA4 [15]. The p.Gly303Trp variant is located within a critical NLS region, adjacent to conserved cysteine residues responsible for zinc coordination. Several pathogenic or likely pathogenic variants have been described in this same domain, including G296S, c.1074delC, c.1075delG, M310V, [15,16], and more recently, the M310T variant [17]. Like Gly303Trp, both M310V and M310T lie within the NLS, a highly conserved region spanning residues 271–322, essential for *GATA4* nuclear import and transcriptional activity. Functional modeling of the M310T variant suggested structural disruption in the NLS and adjacent C-terminal zinc finger domain, and familial segregation analysis revealed consistent transmission with atrial septal defects and arrhythmias across three generations. The proximity of 303 amino acid to 310 methionine further supports the pathogenic relevance of this region. This convergence of evidence reinforces the idea that even single amino acid substitutions within the NLS can significantly impair *GATA4* function and cause congenital cardiac malformations. Notably, the M310V variant has been functionally studied in mouse models, where it was shown to increase the incidence of CHD compared to wild-type controls, highlighting the pathogenic potential of alterations in this specific region. Although the p.Gly303Trp variant has not been previously reported, multiple lines of evidence suggest a likely deleterious effect. It affects a highly conserved residue across vertebrate species, as confirmed by cross-species alignment. In silico prediction tools consistently support a pathogenic impact, with high scores from CADD, REVEL, PolyPhen-2, PaPI, and AlphaMissense, which integrates structural and evolutionary data.

Importantly, AlphaMissense classified the variant as “deleterious (strong)” with a score of 0.999, reinforcing its clinical relevance. Further supporting this notion, Bu and colleagues described a pathogenic M310T variant also located in the NLS domain of GATA4, which segregated with congenital atrial septal defects (ASDs) and arrhythmias in a three-generation pedigree [17]. The parallels between M310T and Gly303Trp, including their spatial proximity and shared evolutionary conservation, strengthen the hypothesis that Gly303Trp may similarly disrupt nuclear import or DNA binding and transcriptional activity. Indeed, this disruption could result from the replacement of the small, flexible glycine (Gly) with the bulkier tryptophan (Trp), potentially altering the structure of the NLS and impairing nuclear import. Additionally, the NLS lies near the C-terminal zinc finger domain, which is crucial for DNA binding and interactions with other transcription factors, and affecting GATA4’s ability to bind DNA and interact with necessary cofactors.

The different phenotypes observed in the two affected family members, a VSD in the son and ASD with PS in the mother, highlight the phenotypic variability often seen in GATA4-related CHD. Such variability may be explained by genetic modifiers, epigenetic factors, or environmental influences [6,18]. Environmental factors such as maternal smoking, diet, and exposure to certain medications, along with epigenetic modifications like DNA methylation and histone modification, may influence the severity and expressivity of GATA4-related CHD [19].

This variability is consistent with emerging evidence in other cardiac genetic conditions, where the same variant or genetic background can result in markedly different clinical presentations, even raising the question of overlapping or distinct pathophysiological mechanisms, as seen in cases of Long-QT syndrome in hypertrophic cardiomyopathy [20]. Moreover, the phenomenon of variable expressivity and incomplete penetrance is well documented for *GATA4* and other transcription factor genes [7]. It was also previously hypothesized that alterations in the NLS domain may interfere with GATA4’s ability to regulate ion channel gene expression or cooperate with co-factors such as NKX2.5 and TBX5, potentially contributing to electrical conduction abnormalities [17]. From a clinical perspective, identifying a rare variant in a gene known to be associated with familial CHD provides important implications for genetic counselling. Molecular diagnosis can help clarify recurrence risks, guide the screening of at-risk relatives, and support early intervention or surveillance in carriers even in the absence of a fully penetrant phenotype. This integrative approach, combining clinical phenotyping with targeted or exome wide sequencing, has also proven effective in other contexts of Mendelian diseases, such as ciliopathies, where genotype phenotype correlations are often complex [21]. Moreover, the expanding recognition of minor and non-canonical genes in cardiovascular genetics further supports the utility of broad sequencing approaches for the diagnosis of familial cardiac conditions, as highlighted in the recent literature on cardiomyopathies [22].

## 4. Materials and Methods

A 38-year-old male was referred to our center for genetic consultation due to recurrent spontaneous abortions in his partner. Peripheral blood samples were collected from all individuals after obtaining informed consent according to ethical guidelines. Genomic DNA was extracted from peripheral blood using the QIAamp DNA Blood Mini Kit, following the manufacturer’s instructions (Qiagen, Hilden, Germany).

Array comparative genomic hybridization (aCGH) analysis was conducted using a 44K platform (Agilent Technologies, Santa Clara, CA, USA) to detect genomic deletions or duplications.

Whole exome sequencing (WES) was performed using the VAHTS Target Capture Core Exome Panel (Vazyme, Nanjing, China), covering 19,441 genes from databases such as RefSeq, CCDS, and ClinVar. Variants detected through WES were annotated and filtered based on functional prediction scores (PolyPhen-2, SIFT, REVEL), disease associations (ClinVar, HGMD, OMIM, GWAS), and population allele frequencies (dbSNP, ALFRED, gnomAD, ExAC, 1000 Genomes), utilizing software enGenome- eVAI v3.5 CE IVD (Pavia, Italy).

The presence of the *GATA4* pathogenic variant was confirmed by Sanger sequencing with a set of primers (F:5′-GGAGAGGGGGATGTTGAGGA-3′, R:5′-CTGGTGCCCTTCTCCAGC-3′). PCR was carried out in 50 µL reactions containing: 1× PCR Buffer (Applied Biosystems, Foster City, CA, USA), 50 µmol/L dNTPs, 1.5 mM MgCl_2_, 1.25 U AmpliTaq Gold polymerase (Applied Biosystems), and 0.2 µmol/L of each primer. The PCR cycling conditions were as follows: initial denaturation at 95 °C for 5 min; 35 cycles at 95 °C for 15 s, 57 °C for 15 s, and 72 °C for 1 min; followed by a final extension at 72 °C for 7 min.

Cycle sequencing was performed using the BigDye Terminator v3.1 Cycle Sequencing Kit (Applied Biosystems), with cycling conditions of 95 °C for 30 s, followed by 35 cycles of 95 °C for 15 s, 50 °C for 15 s, and 60 °C for 4 min. Sequencing products were analyzed on a SeqStudio Genetic Analyzer (Applied Biosystems).

Echocardiographic assessment was performed using standard transthoracic techniques, evaluating ventricular volumes, septal thickness, and pulmonary artery pressures, as detailed in Section 2.

## 5. Conclusions

This study describes the identification of a novel heterozygous missense variant in the *GATA4* gene (c.907G>T; p.Gly303Trp) in a family affected by congenital heart defects, specifically septal anomalies and pulmonary stenosis. The variant lies within a highly conserved region of the C-terminal zinc finger domain, which also encompasses the nuclear localization signal (NLS), a functionally critical region previously implicated in familial CHD. Although the variant is currently classified as a Variant of Uncertain Significance (VUS) according to ACMG criteria, the converging clinical, bioinformatic, and evolutionary evidence, and notably, its identification and segregation in two clearly affected family members presented in this study, strongly support reclassifying this variant from VUS to Likely Pathogenic, even in the absence of additional functional studies. Further functional validation, including protein localization assays, DNA-binding studies, and animal models, will provide additional confirmation of the pathogenic role of the p.Gly303Trp variant and enhance our understanding of its mechanism in cardiac development.

This emphasizes the value of comprehensive genetic and familial analysis in refining variant interpretation in clinical genetics. The different cardiac phenotypes observed among family members highlight the complexity of GATA4-related disease and underscore the influence of genetic background and potential modifying factors. These findings support the inclusion of *GATA4* in diagnostic gene panels for congenital heart disease and emphasize the utility of integrating genetic screening with detailed phenotypic characterization in families with recurrent CHD. Further functional studies and segregation analyses in larger cohorts are needed to fully elucidate the pathogenic role of this variant and its contribution to the spectrum of GATA4-related cardiac anomalies.

## Data Availability

The original contributions presented in this study are included in the article/Supplementary Material. Further inquiries can be directed to the corresponding author.

## Supplementary Materials

The following supporting information can be downloaded at: https://www.mdpi.com/article/10.3390/ijms26104931/s1.
