# Supplementary material for: Characterization of a Novel GATA4 Missense Variant p.Gly303Trp in a Family with Septal Heart Defects and Pulmonary Stenosis
Source: Int J Mol Sci. 2025 May 21;26(10):4931. doi: 10.3390/ijms26104931 (PMC12112125; doi:10.3390/ijms26104931)
Supplement: Supplementary file 1 [file ijms-26-04931-s001.zip › ijms-3622256-supplementary.pdf]

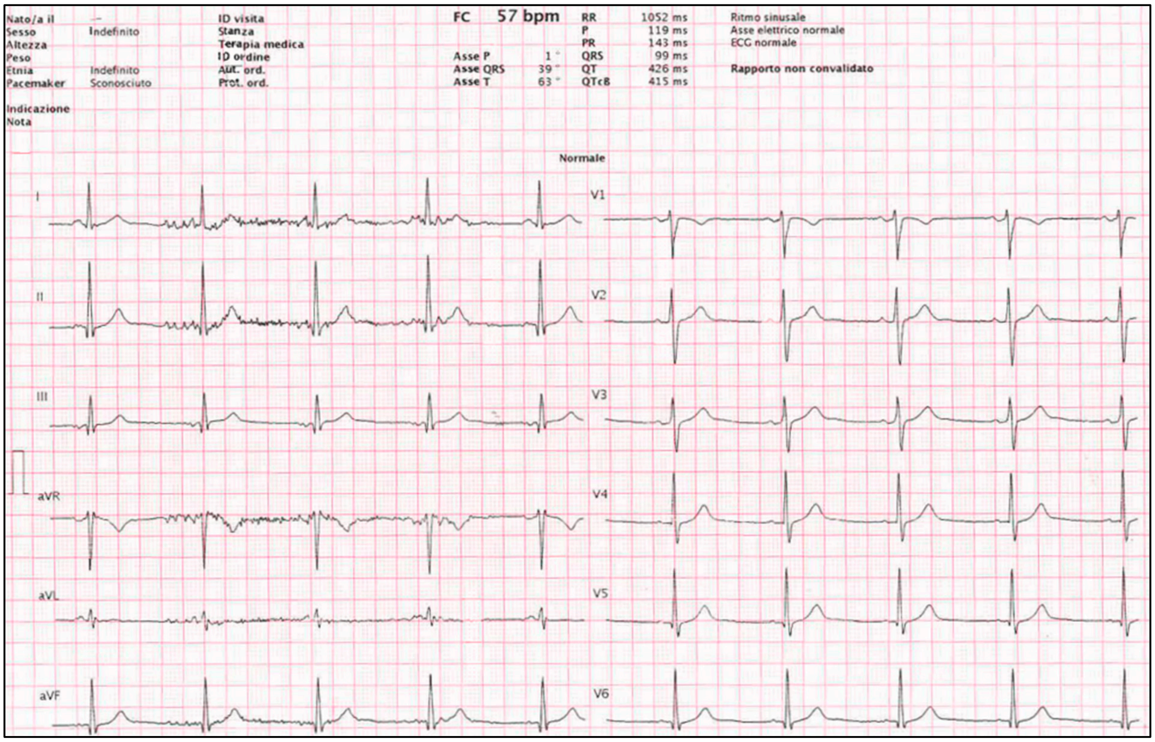

Figure S1. Electrocardiogram (ECG) recording showing sinus rhythm at 57 bpm. The atrioventricular (AV) and intraventricular (IV) conduction are normal, with no evidence of repolarization abnormalities. PR interval: 143 ms; QRS duration: 99 ms; QT interval: 426 ms; QTcB: 415 ms. The tracing confirms a normal electrical axis and no signs of cardiac ischemia or hypertrophy.
